# Ecological influences on the behaviour and fertility of malaria parasites

**DOI:** 10.1186/s12936-016-1271-0

**Published:** 2016-04-18

**Authors:** Lucy M. Carter, Laura C. Pollitt, Laurence G. Wilson, Sarah E. Reece

**Affiliations:** Ashworth Laboratories, School of Biological Sciences, Institute of Evolutionary Biology, University of Edinburgh, Edinburgh, UK; Ashworth Laboratories, Centre for Immunity, Infection and Evolution, School of Biological Sciences, University of Edinburgh, Edinburgh, UK; Department of Physics, University of York, Heslington York, YO10 5DD UK; The Rowland Institute at Harvard, Cambridge, MA 02142 USA; Ashworth Laboratories, School of Biological Sciences, Institute of Immunology and Infection Research, University of Edinburgh, Edinburgh, UK

**Keywords:** Malaria, Transmission, Microgamete, Fertilization, Blood meal

## Abstract

**Background:**

Sexual reproduction in the mosquito is essential for the transmission of malaria parasites and a major target for transmission-blocking interventions. Male gametes need to locate and fertilize females in the challenging environment of the mosquito blood meal, but remarkably little is known about the ecology and behaviour of male gametes.

**Methods:**

Here, a series of experiments explores how some aspects of the chemical and physical environment experienced during mating impacts upon the production, motility, and fertility of male gametes.

**Results and conclusions:**

Specifically, the data confirm that: (a) rates of male gametogenesis vary when induced by the family of compounds (tryptophan metabolites) thought to trigger gamete differentiation in nature; and (b) complex relationships between gametogenesis and mating success exist across parasite species. In addition, the data reveal that (c) microparticles of the same size as red blood cells negatively affect mating success; and (d) instead of swimming in random directions, male gametes may be attracted by female gametes. Understanding the mating ecology of malaria parasites, may offer novel approaches for blocking transmission and explain adaptation to different species of mosquito vectors.

**Electronic supplementary material:**

The online version of this article (doi:10.1186/s12936-016-1271-0) contains supplementary material, which is available to authorized users.

## Background

To achieve transmission between vertebrate hosts, malaria parasites must produce specialized sexual stages (gametocytes). When ingested by a mosquito during a blood meal, gametocytes are activated and rapidly differentiate into male and female gametes (gametogenesis). Microgametes (male gametes) must find and fertilize female gametes within the hostile environment of the blood meal. A series of experiments investigating how the chemical environment influences gametogenesis and mating, and how interactions between microgametes and their physical environments can affect mating success is presented.

### Gametocyte activating factors

In vitro, an increase in pH from ~7.3 to 8 together with at least a 5 °C reduction in temperature initiates gametogenesis [1, 2]. However, while a temperature drop is experienced by parasites moving from the host to the mosquito midgut (in vivo), blood pH does not rise as high as pH8, so other activating factors are thought to be required [3, 4]. Tryptophan metabolites (in particular, xanthurenic acid, XA, and kynurenic acid, KA) have been implicated as natural gametocyte activating factors (GAFs) [5] but there are discrepancies across the literature in the potency of these metabolites at different concentrations and for different species or subspecies [5–8]. Furthermore, the source of these compounds in the host or vector, the identity of their receptors, and how they reach gametocytes in the blood meal remain controversial [6, 9–11]. Also unknown is the relationship between the exflagellation efficacy of each GAF and the resulting reproductive success. There are several reasons to suspect there may not be straightforward (e.g. linear and positive) correlations between GAF concentration and the resulting density of zygotes. First, exflagellation in malaria parasites is rapid and production errors in the formation of anucleate microgametes often occur [2], so the benefits of a GAF that induces a very rapid response could be offset by the costs of increasing this error rate. Second, inhibition of exflagellation at high GAF concentrations has been observed [8]. Third, there may be a trade-off between the quantity of microgametes produced and their quality (e.g. size, or speed, or longevity; all of which are correlated with fertilization success) as has been observed for male gametes in other taxa [12, 13]. Fourth, GAFs may also affect the fertility of female gametes and/or viability of zygotes (ookinetes). These issues are addressed by testing how effective putative GAFs are at inducing exflagellation and quantifying the consequences for mating success and zygote production, across several parasite genetic backgrounds.

### Microgamete behaviour

Once released from the residual gametocyte body during exflagellation, microgametes must locate and fertilize non-motile female gametes in the bloodmeal within a brief (approximately 30 min) time window [1, 2]. It is not clear what determines microgamete lifespan, but many aspects of the environment in the mosquito midgut are assumed to be hostile. A limited supply of resources such as glucose may constrain swimming duration [14–16], host-derived immune factors that are also taken up in the blood meal can kill microgametes or render them infertile, and mosquito immune responses also act on parasites in the blood meal or midgut epithelium [17–25]. The swimming speed of microgametes in red blood cell (RBC)-free medium has been measured at 5 μm/s. No measurements exist for microgametes in vivo, but the maximum (theoretically) possible speed is 50 μm/s [26] based on data from in vitro measurements. This upper limit derives from the constraint that in a viscous medium, such as in a blood meal, microgametes cannot swim faster than the speed at which waves propagate along their flagellum. Even if microgametes travel at the theoretical maximum speed of 50 μm/s, a single microgamete could only explore 1/1000 of the 2 μl mosquito blood meal in 30 min [26]. Given that mating clearly does happen, even when small numbers of gametocytes (<5/μl) are taken up in a blood meal [27], the existence of a mechanism to aid microgametes in their search for female gametes has been proposed [26]. This could involve using interactions with RBCs to increase speed and/or non-random searching of the blood meal.

RBCs in the blood meal readily interact with males undergoing gametogenesis and also with free microgametes. RBCs adhere to males undergoing gametogenesis in a sialic-acid dependent manner to form aggregations called ‘exflagellation centres’ [28]. In theory, physical interactions between RBCs and microgametes could facilitate swimming. A similar phenomenon increases the swimming speed of trypanosomes from 5.7 μm/s in culture medium to 40.0 μm/s in PDMS pillar arrays simulating the RBC arrangement in the host bloodstream [29]. However, any speed increase gained from adhering to RBCs may be negated if RBCs also act as physical barriers that microgametes must negotiate. That RBCs can hinder mating is consistent with the observation that *Plasmodium berghei* ookinete density is lower in *Anopheles* species that concentrate their blood meal the most [30, 31]. Whether interactions with RBC hinder, facilitate, or have a neutral effect, on the ability of microgametes to explore the blood meal in search of females is unknown. To understand the consequences of interactions between microgametes and RBCs, it is necessary to separately study how physical and chemical (e.g. sialic acid) mediated interactions affect mating success. The role of physical interactions is investigated here.

Independent of whether RBCs hinder or facilitate microgametes, non-random searching of the blood meal could occur. It has been proposed that, while in the peripheral circulation of the vertebrate host, gametocytes aggregate to increase the proximity of male and female gametocytes in blood meals [32, 33]. However, this likely results in a trade-off between the probability of a mosquito picking up gametocytes (prevalence) and the number ingested (intensity of infection). Furthermore, it is unclear whether aggregations of gametocytes could remain intact during the feeding process, when anticoagulants are released and the mosquito undergoes diuresis. Thus, an alternative hypothesis is the focus; that microgametes navigate the blood meal non-randomly towards female gametes. This could be achieved using mechanisms such as chemotaxis [34] and/or nanotube-like filaments of gametes (FiGs) [35, 36] to draw microgametes towards female gametes. FiG have been observed on the surface of *Plasmodium falciparum* activated gametocytes and gametes of both sexes [35], but their influence is limited to their length of ~100 μm. Thus, while FiG could operate once microgametes are close to females, their influence is insufficient at the long distances involved in low gametocyte density blood meals. Alternatively, chemotaxis is a common, long-distance, solution to the problem of mate location in externally fertilizing organisms. For example, sea urchin sperm locate eggs by following gradients established from egg secretions [34, 37, 38]. *Escherichia coli* are also capable of following chemical gradients by using simple behavioural rules, for example, by varying run length in response to a chemical gradient [39].

## Methods

Rodent malaria parasites were used to carry out three sets of assays. First, the effect of different GAFs on exflagellation rates and ookinete production, when applied at different concentrations or to different parasite subspecies, was compared. Second, microparticles were used as a proxy for RBCs to investigate whether physical barriers hinder mating and reduce ookinete productivity. Third, whether females can attract microgametes was examined. All procedures were carried out in accordance with the UK Animals (Scientific Procedures) Act 1986.

For all experiments, infections were initiated in male MF1 mice (8–10 weeks old, from an in house supplier, The University of Edinburgh), with 10^7^ parasitized RBCs from donor mice infected with cryopreserved parasites (from The University of Edinburgh’s malaria reagent repository), as detailed below. To examine GAFs, 11 *P. berghei* ANKA infections were used to examine dose-responses; and three *Plasmodium yoelii nigeriensis* N67, three *Plasmodium yoelii subspecies* IV, and five *Plasmodium yoelii yoelii* 17X infections were used to compare the response of different genetic backgrounds. For the microparticle assays, 10 *P. berghei* ANKA and eight *P. berghei* Pb820cl1m1 cl1 (RMgm-164; [40] derived from ANKA) infections were used. To test for microgamete attraction, 13 infections of *P. berghei* ANKA were used. For all experimental infections, mice had been pre-treated with phenylhydrazine at 125 mg/kg (2 days before infection) to enhance the production of gametocytes [41]. The number of independent infections that contributed to each treatment combination are shown in the Additional file 1: Tables 1 to 4, alongside the data for each experiment.

### Pre-assay checks

To verify that there was a sufficient density of mature gametocytes to assay exflagellation and ookinete development on the days that experiments were carried out, preliminary tests were performed on days 4 and 5 post infection for each mouse. This involved culturing 2 μl of tail blood in 100 μl fresh ookinete media (RPMI + 10 % fetal calf serum, pH 8) at 20 °C to stimulate exflagellation [42]. Ten minutes post initiation, 8 μl of this culture was placed under the cover slip of a haemocytometer and the number of exflagellation events observed in 1/9 of the haemocytometer grid (100 nl culture) was recorded. An exflagellation event was defined as a haphazard, rapidly moving parasite extruding flagella; often forming clumps (exflagellation centres) with nearby RBCs. Infections with more than 20 exflagellation events in 1/9 of a haemocytometer were judged suitable for assaying. RBC density counts and thin blood smears were also taken from each infection. Blood smears were stained with Giemsa and the parasitaemia (number of asexual parasites/RBCs) and gametocytaemia (number of gametocytes/RBCs) recorded.

### Gametocyte activating factors

The first experiment compared the efficacy of XA, KA and Tryp at inducing *P. berghei* exflagellation and ookinete production over a wider range of compound concentrations than previously tested. Blood from each infection was used to set up 18 × 200 μl cultures to test three compounds (Xanthurenic Acid (XA), Kynurenic acid (KA) or Tryptophan (Tryp)) at six concentration levels (10^−1^, 10^−2^, 10^−3^, 10^−4^, 10^−5^, and 10^−6^ M). Tryptophan was not expected to be biologically active, but because it is a precursor to the metabolic pathway of XA and KA, it was included to act as another negative control (in addition to pH 7.3). The second experiment tested for genetic variation in exflagellation and ookinete density using three subspecies of *P. yoelii:* (*P. yoelii nigeriensis* N67, *P. yoelii subspecies* IV, and *P. yoelii yoelii* 17X) when cultured in 10^−4^M XA, KA or Tryp. Analysis of the sex ratio (proportion of male gametocytes) from smears of the blood used to initiate cultures found no significant difference across subspecies (F_2,8_ = 1.42, P = 0.296). For both experiments, cultures were set up with RPMI, 10 % foetal calf serum and the specified type and concentration of GAF. The pH of each culture was adjusted to 7.3 after the addition of compounds to prevent any variation in pH from confounding results. A pH of 7.3 was used as a control because in our lab, the commonly used “negative control” condition of pH 7.4 – even in the absence of chemical GAFS - resulted in significant exflagellation, whereas pH 7.3 did not (see Additional file 1: Figure 1). Furthermore, pH 7.3 provides an environment more representative of infected blood than pH 7.4 [43]. Negative (pH 7.3) and positive (pH 8) control cultures were always set up from the same infections as cultures containing GAFs.

Tail blood was collected from infected mice, added to the pre-prepared cultures, and vortexed. For *P. berghei* cultures, 2 μl infected tail blood was cultured in 200 μl ookinete media, but only 100 μl media was used for *P. yoelii* cultures because *P. yoelii* gametocyte density is on average 10-fold lower than for *P. berghei* (in these experiments there was a mean of 1.35 × 10^7^ gametocytes/ml blood for *P. y . yoelii*, *P. y. nigeriensis,* and *P. yoelii subspeciesvs* 1.74 × 10^8^ gametocytes/ml blood for *P. berghei*). Increasing the gametocyte density in *P. yoelii* cultures enabled faster and more efficient exflagellation assays, and a 1 % increase in the density of blood in the culture does not interfere with fertilization or ookinete maturation. After 14 min, cultures were subsampled and exflagellation was recorded using a haemocytometer (as described above). If fewer than 30 exflagellation events were observed in 1/9 of the haemocytometer area, then exflagellation density was counted over 1/3 of the haemocytometer area. Exflagellation density was counted over the whole of the haemocytometer area for the negative control cultures (pH 7.3). All cultures were placed in an incubator (at 20 °C for *P. berghei* and 24 °C for *P. yoelii* spp) for 18–21 h, to allow for fertilization and ookinete maturation. Following this incubation period, cultures were removed from the incubator and vortexed for 20 s to eliminate clumping of ookinetes and female gametocytes, and ookinete density was assayed over 2/3 of the haemocytometer area.

### Microparticle density

This experiment used microparticles (poly-methyl methacrylate-particles, microparticles GmbH, diameter 6.33 ± 0.13 μm) as a proxy for RBCs to test whether they can act as physical barriers to microgametes, resulting a reduction in ookinete production. *P. berghei* infected blood was collected via cardiac puncture from anaesthetized mice (total blood volume collected ranged from 0.5 to 1.6 ml) and added to 10 ml of gametocyte stasis media (RPMI + 5 % foetal calf serum, pH 7.3, at 37 °C). The culture was then immediately passed through a magnetic column (MACS LS separation columns, Miltenyl Biotech) to which the haem–rich magnetic gametocytes adhered, whilst the serum, asexual and uninfected RBCs passed though. Then, 3 ml gametocyte stasis media was added to wash any remaining non–gametocyte stages (non-magnetic) from the column. The column was then removed from the magnet; 1 ml ookinete media was added and immediately forced through the column to flush out the gametocytes into a sterile centrifuge tube. This was repeated three times, resulting in 3 ml of purified gametocyte culture. The gametocytes were then spun at 2500 rpm for 3 min at 37 °C, the supernatant removed, and 3 μl from the remaining pellet of pure gametocytes was aliquoted into pre-prepared ookinete media (total volume: 23 μl, RPMI + 10 % foetal calf serum, pH 8, 20 °C) containing different ratios of microparticles: media, according to the four treatment groups detailed below and in Table 1. After washing the microparticles in ookinete media they were suspended ~100 μg in 20 μl ookinete media to produce stock solution. Four different culture conditions were prepared from the stock solution with increasing densities of microparticles: 0 % (newtonian fluid control), 1, 35, and 60 %, to represent the range of RBC densities encountered in routine culture, host blood, and the post-diuresis blood meal. For each culture in the 1 % treatment, 0.2 μl microparticle stock solution was added to 19.8 μl ookinete media; for the 35 % treatment, 8 μl microparticle stock solution was added to 12 μl ookinete media; and for the 60 % treatment, 14 μl microparticle stock solution was added to 6 μl ookinete media.

**Table 1:** Summary of treatments and rationales for the microparticle experiment

Microparticle concentration (%)	Rationale
0	Absence of microparticles (~Newtonian fluid)
2	Microparticle density similar to RBC density in routine ookinete culture
35	Microparticle density similar to RBC density in healthy host blood
60	Microparticle density similar to RBC density in a blood meal after diuresis

Tail blood cultures were also set up from the same infections to confirm that the gametocytes successfully fertilized and developed into ookinetes in routine culture conditions. Pilot experiments showed that microgametes do not form exflagellation centres with microparticles, confirming that any RBC-microgamete interactions do not involve simple electrostatic attraction to the hydrophilic RBC coat [28]. While the ratio of media to microparticle solution varied across the different microparticle densities, the total volume of cultures was kept constant. To investigate whether media (i.e. resource) limitation at high microparticle densities could negatively affect ookinete development (and thus, confound the effect of microparticles), an additional 80 μl ookinete media was added to half of all cultures across all treatment groups 2 h after culture initiation (i.e., once fertilization was complete). All cultures were incubated at pH 8 and at 20 °C for 18–21 h before a haemocytometer was used to calculate the total number of ookinetes/ml blood. Some cultures were diluted before counting to avoid missing ookinetes masked by high densities of microparticles.

### Microgamete attraction

**Fig. 1:**
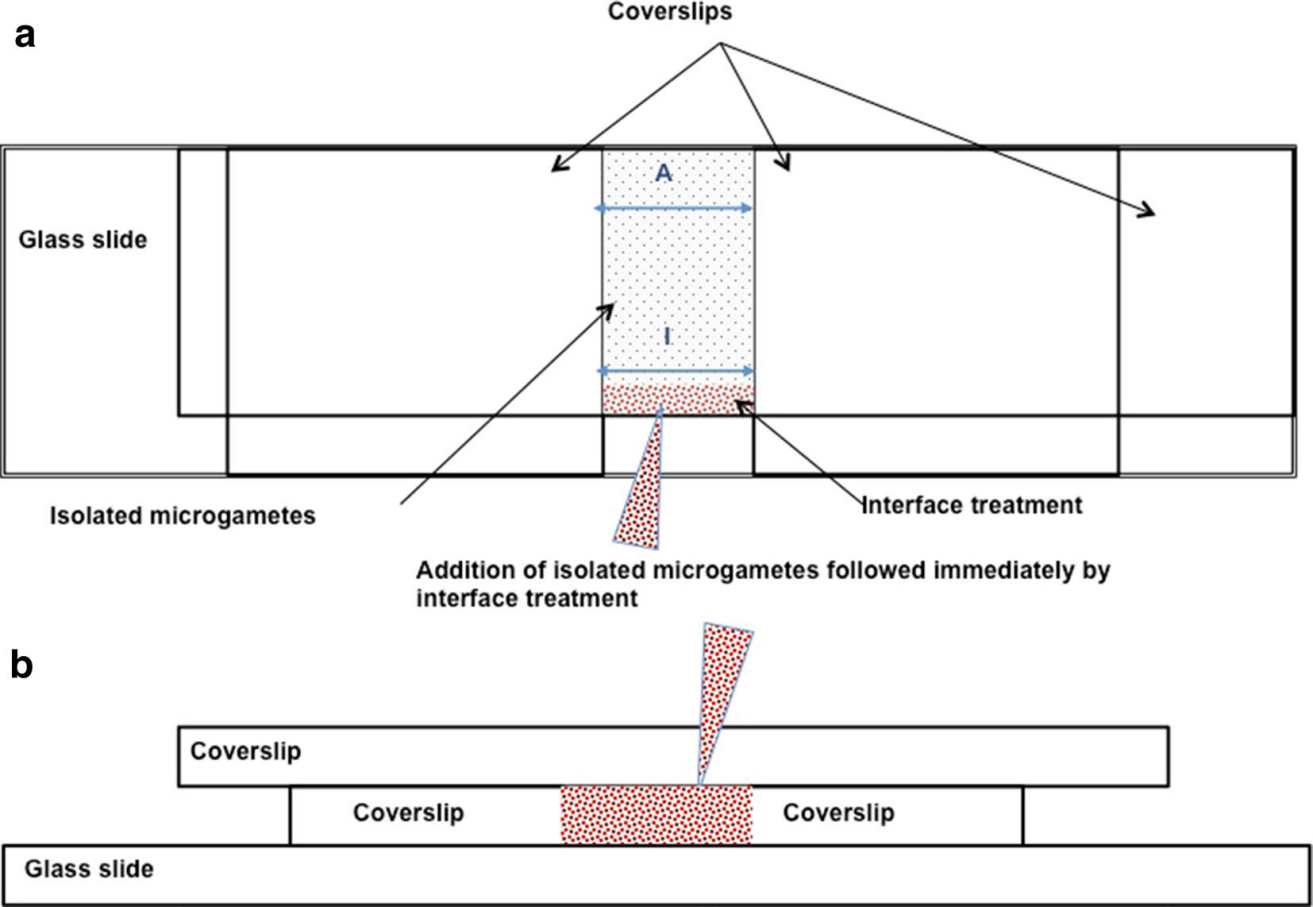
Chamber design and assay set up for the chemotaxis experiment. Three coverslips (1 *large rectangular* and 2 *small* and *square*) were fixed on to each slide to create a chamber at the centre. For each assay, 7 µl microgamete culture was placed into the chamber, followed immediately by 2 μl of the treatment to create an interface. “*A*” represents the area counted for the away location, and “*I*” for the interface location. **a** View from *above*, **b** view from the *side* (not to scale)

This experiment tested whether *P. berghei* microgametes preferentially aggregate in regions containing material from females, when compared to regions containing material from RBC or asexual stages. Glass chambers were constructed using slides, coverslips fixed with optical adhesive (Norland) and UV light, as detailed in Fig. 1, to create an assay environment without ‘flow’. This means that all microgamete movement is due to Brownian motion plus their own motility. For each assay, microgametes were isolated from 20 μl infected blood, placed into 20 μl ookinete media (RPMI + 10 % fetal calf serum, pH 8, 20 °C) for 20 min (to allow sufficient time for the activation of male gametocytes, exflagellation and release of microgametes), and then spun down at 2000 rpm for 1 min to produce a supernatant containing purified microgametes. 7 μl of the supernatant was placed in the test chamber, immediately followed by 2 μl of the cue treatment material, as illustrated in Fig. 1. This arrangement creates two distinct, separate regions within the sample chamber: inside or at the interface with the cue treatment (“I”) and at least 12.5 mm away (“A”) from the interface. The probability that any particles (i.e., cue material and cells) placed at the interface had diffused into the ‘A’ location within 20 min—the total duration of the experiments—is less than 6 × 10^−13^ assuming typical molecular diffusivity, no edge effects, and that the distance along the chamber is the only relevant quantity (i.e., concentration is constant as a function of channel width and height) [44].

If microgametes are chemotactically attracted by the cue treatments, they are expected to accumulate in the cue treatment region (“I”), while numbers in the unaffected region far from the interface (“A”) act as a control. The distance from sample point A to the interface is significantly larger than the distance that a microgamete can swim in 20 min, and so measurements at point A control for variation in the number of motile gametes in the sample. Immediately (t = 0) after placing microgametes on the slide the number of microgametes/field (microgamete density) was recorded at points I and A. The location counted first was randomly selected and counting in each location was limited to a duration of 3 min. Thus, for the t = 0 time point, counts were taken over a range of 0–6 min post assay initiation. 20–26 min after adding microgametes to the slide (t = 20), microgamete density counts were taken again at both locations, starting with the location that the t = 0 counts were made first. For example, for assay *x*, microgamete density was counted at the interface with the cue treatment (I) from 0–3 min, and away from the interface (A) from 3–6 min, then from 20–23 min post assay initiation, microgamete density was counted at (I), and then (A) from 23–26 min. As microgametes had spent 20 min in ookinete media prior to being placed on the slide, the following 20 min time period of the assay reflects the period in which fertilization is thought to occur in the blood meal [1, 2].

Microgametes were assayed in arenas containing five different cue treatments (Table 2): uninfected RBCs; live asexuals; lysed asexuals; live female gametes; and lysed female gametes. The experiment was designed so that the responses to all cues could be compared to each other, or combined to test for general responses according to the classifications outlined in Table 2. Each ‘live’ treatment was freshly prepared and ‘lysed’ cues were prepared in advance. The cue of uninfected RBCs (RBC) was prepared from 20 μl tail blood, from a naive mouse, placed in 20 μl ookinete media and centrifuged at 10,000 rpm for 2 min. The supernatant (serum) was removed leaving uninfected RBCs. The other four cues were prepared from infected blood, as follows. 0.5–1.6 ml blood was collected from anaesthetized *P. berghei* infected mice by cardiac puncture and added to 10 ml of ookinete media at 20 °C for 20 min to exflagellate.

**Table 2:** Summary of treatments, number of independent infections, rationales, and classifications in the chemotaxis experiment

Treatment	N	Rationale	Description
Uninfected RBC	10	Control. To test whether microgametes are attracted to a zone of cells, regardless of cell type	Fresh blood
Live asexuals	13	Control. To test whether microgametes are attracted to parasites/parasitized RBC	Purified asexual- stages in ookinete media
Lysed asexuals	12	Control. To test whether microgametes are attracted to material liberated from lysed parasites	Asexual–stage parasites were cultured in ookinete media for 3 h before lysis (to match conditions used to prepare the lysed female treatment group)
Live females	9	Female. To test if microgametes are attracted to live female gametes	Purified female gametes in ookinete media
Lysed females	10	Female. To test if microgametes are attracted to material liberated from lysed females	Female gametes cultured in ookinete media for 3 h to allow for the production and secretion of chemotactic cues as well as all other cell contents, before being lysed

The analysis involved comparing the effect of individual treatments as well as comparing treatments grouped into ‘control’ versus ‘female’

Microgametes were separated from other cells by passing through a magnetic column (MACS, Miltenyl Biotech) to which the female gametes and any remaining non – activated male gametocytes adhered. 3 × 3 ml washes of ookinete media were then passed through the column to wash the uninfected RBC and asexual stage parasites out, along with microgametes. The flow through was collected and spun at 10,000 rpm for 2 min. The supernatant (serum and media) was removed; leaving a pellet of asexual and uninfected RBC, which formed the ‘live asexual’ treatment group. Aliquots of this pellet were used to prepare the ‘lysed asexuals’ treatment group via three rounds of freeze-thaw at −80 °C and 37 °C, before storage at −80 °C. This method completely lyses parasite infected RBCs [45].

After the last wash, the column was removed from the magnet and 3 × 1 ml ookinete media was added to flush out the activated female gametes and gametocytes. This culture was then spun at 2500 rpm for 3 min at 37 °C. The supernatant was removed and the remaining pellet of pure female gametes was resuspended in 1 ml of ookinete media containing aphidicolin (Sigma-Aldrich, UK) at a concentration of 5 × 10^−4^ M, and incubated at 21 °C for 10 min. Aphidicolin irreversibly prevents any remaining males from forming microgametes, that could confound the behaviour of the test microgametes (e.g. [22]). The culture was spun and the pellet washed with ookinete media to remove the aphidicolin, leaving a pellet in which the functional cells consist of activated female gametes. The females were then aliquoted into two tubes; one for use for the ‘live female’ treatment assay, and the second to form the ‘lysed female’ treatment. This second tube was incubated at 20 °C for 3 h to allow the female gametes to produce and release any potential chemotactic cues before lysis, as described above for the ‘lysed asexuals’ treatment.

### Analyses

All data were analysed using R version 3.0.2. All models included ‘infection ID’ as a random effect to control for multiple cultures being initiated from each mouse, thereby avoiding pseudoreplication. For all experiments, models were minimized following stepwise deletion of the least significant term and using log likelihood ratio tests (χ^2^) to evaluate the change in model deviance, until only significant terms remained in the model. Linear mixed effects models (LME) were used to analyse the GAF data after exflagellation density (exflagellation/ml blood) and ookinete densities (ookinetes/ml blood) were adjusted for dilution factors. Negative control cultures (pH 7.3) were not included in the models, because there was no equivalent variation in “concentration” to compare with the GAF treatments (i.e. it could not be included in an interaction with concentration). Exflagellation density for each GAF, concentration, and parasite genetic background, relative to the pH 8 control, is reported. Ookinete density for each GAF, concentration, and parasite genetic background, relative to the pH 8 control, is reported, except for *P. yoeliisubspecies* where no ookinetes were formed in pH 8 cultures, so unadjusted ookinete densities were analysed. All response variables were Log2 transformed (+0.001 for *P. yoelii* exflagellation densities), to conform to the assumptions of normality. Zero counts were removed before anlaysis to minimize problems of zero inflation skewing the error distribution. Post hoc Tukey tests were carried out to test for differences between individual treatment groups. The microparticle data was analysed in the same way following Log10 transformation. The microgamete attraction data were analysed using generalized linear mixed models (GLMM) because response variables were strictly bounded (between zero and one), had non-constant variance and non-normal errors. GLMMs with a binomial error structure and a logit link function were fitted to test whether there were any changes over time between cue treatments in the proportion of microgametes observed at the interface. This approach accounts for any other changes in the chamber environment over time, makes full use of all the data collected and weights the analysis based on the density of microgametes observed at the start of the assay. GLMMs were constructed with the binomial response variable as the proportion of the total microgametes at the interface (I_md_/(I_md_ + A_md_)), where ‘I_md_’ is the microgamete density at the interface and ‘A_md_’ is the microgamete density away from the interface. Explanatory variables included the assay time (either t = 0, or t = 20) and the cue interface treatment.

## Results and discussion

### Gametocyte activating factors

To investigate how effective putative GAFs are at inducing exflagellation in vitro*, P. berghei* exflagellation rates were quantified over a wider concentration range of XA and KA than previously examined and the effect of tryptophan (the precursor for XA and KA) was tested for the first time. To test for genetic variation in responses to GAFs, exflagellation rates were compared for three subspecies of *P. yoelii* (*P. yoelii nigeriensis*, *P. yoelii yoelii* and *P. yoelii subspecies*) when exposed to 10^−4^ M XA, KA and Tryp. By following each culture from exflagellation to ookinete production, the experiments reported here more closely connect gametocyte activation to reproductive success (ookinete density) than previous studies. No exflagellation was observed in cultures with GAFs at 10^−1^ M so these data are excluded from analysis.

**Fig. 2:**
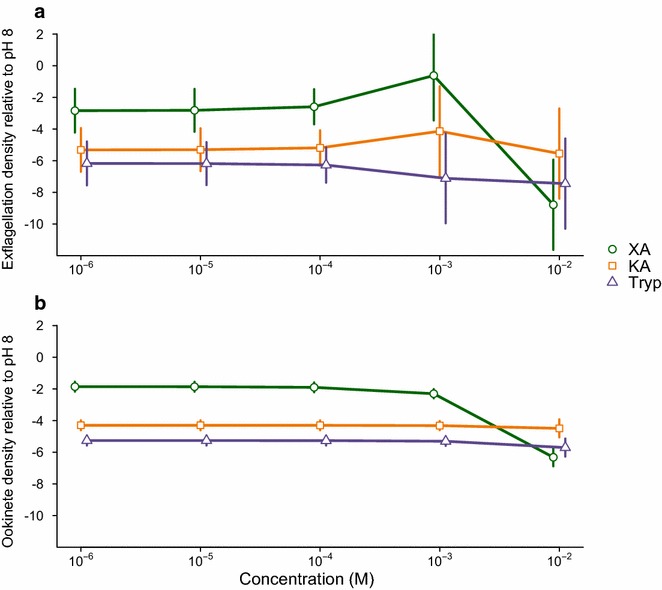
Dose response to GAFs. Mean ± SEM of log2 transformed densities of exflagellating males (**a**) and ookinetes (**b**) relative to the pH 8 control, when exposed to 10^−6^ to 10^−2^ M xanthurenic acid (XA), kyneurenic acid (KA), or tryptophan (Tryp). n = 10–11 (independent infections) for each GAF and dose combination

*Plasmodium berghei* exflagellation rates follow variable patterns accross the different GAF concentrations (Fig. 2a, GAF*concentration: χ_2,8_^2^ = 17.74, P < 0.0001, Additional file 1: Table 1). In agreement with previous studies [5–7], XA is the most potent GAF. At its peak of 10^−3^ M XA induces >6-fold more exflagellation than KA and >11-fold more than Tryp, but has an inhibitory effect at the highest concentration. That Tryp induced exflagellation was unexpected, but it was the least potent GAF. Little variation in exflagellation occurs in response to KA and Tryp over the range of concentrations tested, though there is a slight trend for inhibition at the highest concentrations. In the range of 10^−3^ to 10^−5^ M, XA is just as potent as pH 8 but KA and Tryp induced less than 50 % of that by pH8. Overall, the results are consistent with observations that XA at ~10^−3^M induces more exflagellation than other GAFs, though KA induces less exflagellation than previous studies [5]. Discrepencies may be due to differences in culture set up; in particular, the slightly lower pH used here.

Ookinete densities also varied depending on the presence and concentration of XA, KA and Tryp (Fig. 2b, GAF*concentration: χ_2,6_^2^ = 26.81, P < 0.0001, Additional file 1: Table 1). At low and intermediate concentrations (10^−6^ to 10^−4^ M), XA produced the highest density of ookinetes (~3-fold more than KA and Tryp). The shape of the dose-patterns are generally similar to those for exflagellation, with XA having an inhibitory effect at 10^−2^ M, little variation in ookinete densities across the gradients for KA and Tryp, and KA producing slightly higher ookinete densities than Tryp. However, whilst XA at 10^−3^ M induced the highest exflagellation rate, ookinete density starts to reduce at this concentration. If all microgametes resulting from the GAFs were equally able to mate and/or produce viable offspring, then ookinete densities (Fig. 2b) would more closely match the patterns for exflagellation in Fig. 2a. The 6-fold higher (on average) exflagellation rate induced by XA compared to the other GAFS only translated to 3-fold (on average) greater ookinetes, suggesting there may be either negative effects of XA, or positive effects of KA and Tryp, on mating success or zygote development. Furthermore, the data suggest that using XA at 10^−3^ M maximizes exflagellation [5–8] but ookinetes are maximized at lower concentrations of XA. This may be due to toxicity to females or zygotes, or a density dependent trade-off between quantity and quality of microgametes.

**Fig. 3:**
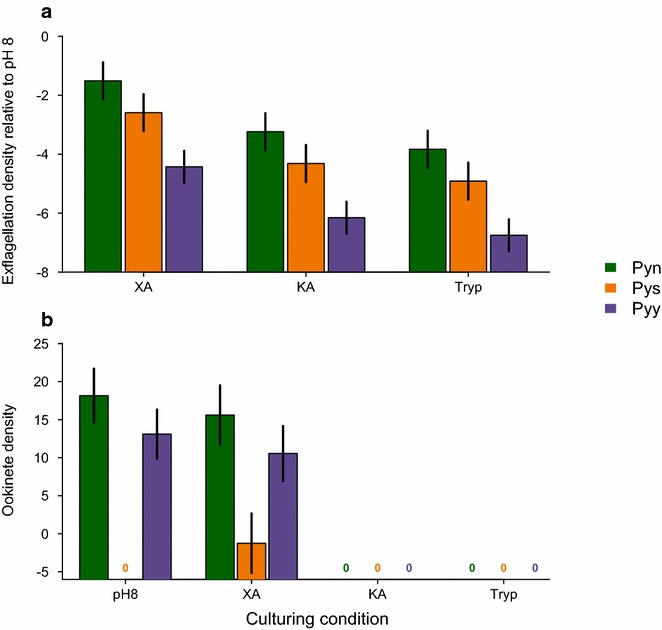
Variation in response to GAFs of *Plasmodium yoelii* subspecies. Responses of *P. yoelii* subspecies (Pyn: *P. yoelii nigeriensis*; Pys*: P. yoelii subspecies;* and Pyy: *P. yoelii yoelii)* to GAFs at 10^−4^ M (XA: xanthurenic acid; KA: kynurenic acid; and Tryp: tryptophan). **a** Mean ± SEM log2 + 0.001 transformed densities of exflagellating males relative to the pH 8 control. **b** Mean ± SEM log2 transformed densities of ookinetes. *Zeros* indicate combinations where no ookinetes were observed. N ranges from 3 to 5 independent infections for each GAF

The effect of each compound on exflagellation did not significantly differ between any of the *P. yoelii* subspecies (compound*subspecies: χ_4,7_^2^ = 4.91, P = 0.297, Additional file 1: Table S2). However, the three subspecies (χ_2,5_^2^ = 19.31, P < 0.001) showed significant differences in their exflagellation responses to the three GAF types (χ_2,5_^2^ = 13.26, P = 0.001) (Fig. 3a). Exflagellation rates were higher for *P. y. nigeriensis* and *P. yoelii subspecies* than observed for *P. y. yoelii* (~2-fold lower). The response to GAFs followed the same pattern as for *P. berghei:* with the highest exflagellation in XA cultures and lowest in Tryp cultures. No ookinetes were observed in any cultures containing KA or Tryp across all subspecies, or for *P. yoelii subspecies* at pH 8. Zero ookinete counts for KA and Tryp may be due to post exflagellation inhibitory effects on the microgametes, female gametes, and/or zygotes, suggesting *P. yoelii* is more sensitive to inhibition than *P. berghei*.

Comparing the cultures in which ookinetes were observed revealed a different pattern to exflagellation. *P. y. nigeriensis* produced the most ookinetes (as for exflagellation) but *P. yoelii subspecies* produced the least (χ_2,3_^2^ = 16.61, P < 0.0001, Fig. 3b, Additional file 1: Table 2). However, within each subspecies (except *P. yoelii subspecies*), there was no significant difference between ookinete densities in pH 8 and XA cultures (GAF*subspecies: χ_2,6_^2^ = 4.63, P = 0.099 and GAF: χ_1,5_^2^ = 0.91, P = 0.340, Fig. 3b). The different responses of the *P. yoelii* subspecies provide evidence for genetic variation on which selection can act to facilitate evolution in response to GAFs. For example, differential exposure to XA/KA/Tryp across mosquito species could select for adaptation to a specific vector species. Further characterizing the extent of genetic variation in sensitivity to GAFs (as well as subsequent ookinete yields) is required to improve understanding of the selective forces shaping mating success.

### Microparticle density

**Fig. 4:**
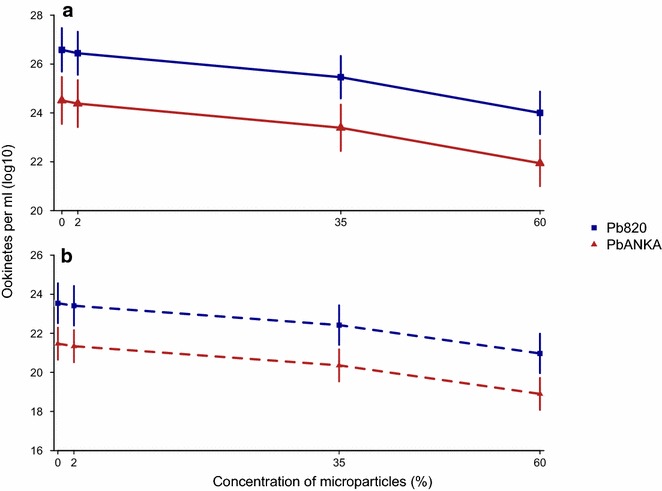
The density of microparticles affects mating success as measured by ookinete density. Mean ± SEM log10 transformed ookinetes/ml blood for each *P. berghei* line (Pb820 and PbANKA) in cultures in which media was replenished post-fertilization (**a**), or not replenished (**b**). N ranges from 3 to 6 independent infections for each mean

To examine whether the physical presence of RBCs hinder microgametes by, for example, acting as barriers to motility, biocompatible particles (‘microparticles’) were used. Microparticles are similar to the size, shape and hydrophilic surface of murine RBCs but without the sialic acid surface coat (allowing the effect of physical interactions to be examined). The effect of adding microparticles at different densities to cultures of purified *P. berghei* gametocytes on ookinete density, as a measure of reproductive success, was examined. Whether replenishing media after fertilization increased ookinete yield was also tested. The effect of microparticle density on ookinete density (Fig. 4) was not dependent on whether media was replenished post fertilization (microparticle density*media: χ_3,8_^2^ = 6.76, P = 0.08), but there were significant (main) effects of microparticle density (χ_3,5_^2^ = 36.74, P < 0.0001) and media replenishment (χ_1,7_^2^ = 8.33, P = 0.04). Increasing microparticle concentration from 0 to 60 % resulted in a 2- to 10-fold decrease in ookinete yield, depending on the parasite line and whether media was replenished (Table S3). Replenishing media post fertilization increased ookinete density by 12 %. Furthermore, whilst both *P. berghei* lines were affected by microparticles and media replenishment in the same way, *P. berghei* ANKA ookinete densities were 9 % lower overall than *P. berghei* 820 ookinete densities (Fig. 4; χ_1,7_^2^ = 4.25, P = 0.04).

How microparticles at high densities interfere with mating is unknown but the most parsimonious explanation is that microparticles are physical barriers to microgamete movement. Assuming microparticles are a good model for the physical characteristics of RBC (e.g. similar packing arrangement and hydrophilic surface), this could explain why ookinete density is lower in vector species that use diuresis to concentrate their blood meals, in which up to 55 % of the fluid ingested can be excreted [46]. Vector control measures that cause parasites to encounter novel vector species (as is occurring due to insecticide use) [47] with different diuresis behaviours could, therefore, affect transmission. Host anaemia could further complicate an interaction between transmission success and diuresis. Whether the cost of RBC acting as a barrier to motility can be compensated for by any benefits from chemical interactions between microgametes and RBC (e.g. adhesion to sialic-acid), or whether chemical interactions are also costly, now needs to be investigated. Across all microparticle densities, ookinete density was higher when media was replenished. Media replenishment could not affect mating success (because replenishment occurred post fertilization), so must affect the ability of zygotes to transform into ookinetes. This suggests that media replacement overcomes some limitation(s) in the post-fertilization environment. This could include re-stabilizing pH, and/or diluting out toxins produced by immune cells or ruptured RBC, and/or replenishing nutrients/resources. Fortunately, for studies of fertilization and ookinetes, media replenishment is a simple technique to maximize yields.

### Microgamete attraction

**Fig. 5:**
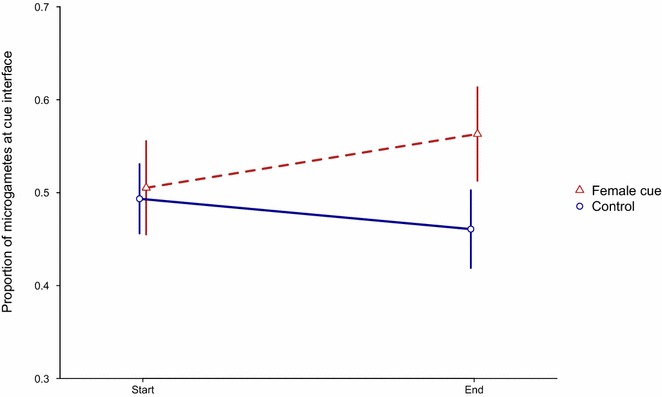
Microgametes move towards a female cue. The mean proportion of microgametes (±95 % confidence interval) at the interface with either a control treatments (RBC and/or asexual stage material) or female material treatments (live female gametes or lysed female gametes) at the start of the assay (0–6 min), or after 20–26 min in the chamber (End). Note that 95 % CI are given instead of SEM for clarity of the borderline difference. N = 3 independent infections

To investigate whether microgametes swim in random directions or preferentially move towards females, whether microgametes were attracted or repelled by material (cues) generated from: live female gametes, lysed female gametes, live asexuals, lysed asexuals, and uninfected RBCs was tested. There was no significant difference in microgamete location when comparing all five cue treatments individually (χ_4,7_^2^ = 7.82, P = 0.098, Table S4), or when comparing live female gametes to lysed females (χ_2,9_^2^ = 0.19, P = 0.907), or live asexuals to lysed asexuals (χ_2,7_^2^ = 2.33, P = 0.313). Therefore, the analysis was simplified by combining cues into three categories: “RBC”, “asexuals” and “females”. There was no significant difference in the location of microgametes exposed to “RBC” and “asexuals” (χ_2,5_^2^ = 1.81, P = 0.404) and so the cue treatments were simplified further, into two categories: “control” (RBC, live asexuals and lysed asexuals) and “females” (live and lysed). There was a borderline significant trend for microgametes to accumulate at an interface with “females” and be repelled from an interface of “control” cues (Fig. 5; χ_1,5_^2^ = 3.83, P = 0.051). Specifically, after 20 min, the proportion of microgametes at an interface with female material increased by 11 % (from 0.50 to 0.56), and reduced by 6 % at an interface with control material (from 0.49 to 0.46). Whilst this difference of 17 % is of borderline statistical significance, it suggests that female gametes attract males and that microgametes search the blood meal non-randomly to find mates.

In general, conclusively demonstrating chemotactic behaviour is extremely difficult. Chemotaxis in other species often manifests itself as a statistical effect, in which cells bias random-walk search processes; this offers some degree of robustness in search strategy, against the randomizing effects of rotational Brownian motion [48]. The short time in which microgametes must find female gametes limits the opportunity for ‘wrong turns’ in their search procedure, but their motion nevertheless appears somewhat erratic and plausibly constitutes a random walk of runs and reversals [26]. Furthermore, the relatively small number of microgametes that can be collected to assay in each sample naturally results in noisy data. This is further compounded by the short distance travelled by an individual microgamete within its active phase, allowing it to explore only a limited local environment. The short life span of microgametes also prevents long-time average measurement of chemotactic behaviour which can be helpful in observing small, but critical effects.

Our cue treatments were designed simply to test whether a response could be elicited in microgamete location, rather than to identify precisely what they detect. Microgametes could be attracted up a gradient of chemoattractant released by the activated female gametes (chemotaxis), in a similar mechanism to bacterial food searching [49]. Gradients could readily form in the blood meal because they consist of a viscous medium in a small volume. The forces generated by peristalsis during diuresis are unlikely to generate a well-mixed blood meal because the flow rate required to generate sufficient turbulence would need to be in the order of metres per second. The time-limited nature of exflagellation and fertilization suggests that any female-derived chemo-attractants are most likely to be released immediately upon egress of the female gamete from the residual gametocyte. Gamete egress is known to be facilitated by a local increase in calcium ions (Ca^2+^), activation of calcium dependent protein kinases and the localization of osmiophilic bodies at the plasma membrane [50, 51]. Osmiophilic bodies release their contents into the parasitophorous vacuole which then becomes released into the environment upon egress from the RBC [52].

Identifying whether a chemoattractant is released from osmiophilic bodies (or simply whether osmiophilic bodies are involved) could be facilitated by comparing microgamete motility at interfaces with activated wild type female gametocytes and Pfg377-KO female gametocytes which lack the full complement of osmiophilic bodies [42]. Likewise, testing the effect of different Ca^2+^ concentrations (at an interface) on microgamete motility could provide clues as to whether microgametes use Ca^2+^ as a cue for the presence of activated female gametes. Exposing each batch of microgametes to multiple cues simultaneously and allowing more time for them to change location before assaying could also help determine how microgametes discriminate between cues. In addition, ‘optical tweezers’ are one tool that could be used to measure the force of attraction between isolated microgametes and female gametes vs. uninfected RBCs [53, 54]. Advances in microscopy and microfabrication, often originating from physics laboratories, are ideally suited to addressing these questions. If a chemoattractant is important for fertilization in malaria parasites, then disrupting it could offer a novel approach to blocking transmission. For example, the egg-derived tryptophan signal that attracts male sea urchin sperm is extinguished by the addition of the enzyme tryptophanase [38].

## Conclusions

Developing drugs and/or vaccines that prevent transmission of *Plasmodium* species by disrupting gametogenesis and mating are major goals of biomedicine. Understanding how environmental factors are involved in initiating gametogenesis, and their influence on mating behaviour, is central to predicting how parasites will evolve in response to transmission blocking interventions. Furthermore, interfering with GAF and chemotaxis detection/response systems could offer novel targets for intervention. Understanding the impact of parasite interactions with RBC on reproductive success could also be important in the context of adaptation to mosquito vectors.

## Additional file

10.1186/s12936-016-1271-0 Additional methods and results for the pH of ookinete cultures and the raw data from each experiment.

## Abbreviations

GAF: gametocyte activating factor
RBC: red blood cell
XA: xanthurenic acid
KA: kynurenic acid
Tryp: tryptophan
FiG: filament of gametes
