# Supplementary material for: Ecological influences on the behaviour and fertility of malaria parasites
Source: Malar J. 2016 Apr 18;15:220. doi: 10.1186/s12936-016-1271-0 (PMC4835847; doi:10.1186/s12936-016-1271-0)
Supplement: Supplementary file 1 — 10.1186/s12936-016-1271-0 Additional methods and results for the pH of ookinete cultures and the raw data from each experiment. [file 12936_2016_1271_MOESM1_ESM.docx]

**ADDITIONAL FILE:**

**pH of control cultures**

Background & Approach**:** Accurately testing the responses of parasites to GAFs requires that no additional culture characteristics – such as pH – confound responses to compound doses. Raising the pH above that of blood readily induces exflagellation *in vitro* (Carter and Nijhout, 1977, Sinden et al., 2010). A pH of 7.4 in culture media is often used to inhibit exflagellation, but this is slightly higher than that of blood (pH 7.25-3) (Chang et al., 2001). Therefore we carried out a pilot experiment to determine whether pH 7.3 or 7.4 is more effective at inhibiting exflagellation in control conditions of *in vitro* cultures. We used 3 independent infections of *P. yoelii yoelii*, strain 17X and cultured 2 μl tail blood in 100 μl media (containing RPMI + 10% foetal calf serum, at 20 ^o^C) at either pH8, pH 7.4, pH 7.3 or 10^-4^M XA (at pH 7.3) with *P. yoelii yoelii* infected tail blood and exflagellation assays were carried out as described in the main text. Each infection contributed a sample of parasites to each treatment.

Results & Conclusions: Culture conditions significantly affected exflagellation rates (χ^2^_3,6_= 33.791, p<0.001) (Figure SI.1). Post hoc Tukey tests revealed no significant difference between responses to pH 8 and 10^-4^M XA. While pH 7.4 (0.09 ± 0.04) induced less exflagellation than pH 8, its effect was not significantly different to 10^-4^M XA (0.21 ± 0.09). Only pH 7.3 (0.01 ± 0.006) induced significantly less exflagellation than 10^-4^M XA. Therefore, because only pH 7.3 significantly inhibited exflagellation we deemed it more appropriate as a negative control than pH 7.4.

**Figure 1.** Mean ±SEM proportion of males that underwent exflagellation in response to cultures pH 8, 10^-4^M XA, pH 7.4, and pH 7.3 (relative to the total number of males exflagellating from each infection). N=3 independent infections.

**Gametocyte activating factors experiment, raw means.**

**Table 1. *P. berghei* ANKA** raw data. These data were log2 transformed prior to analysis, as displayed in figure 2.

| **Treatment** | **Concentration** | **n** | **Mean exflagellation density relative to pH8** | **SEM** | **Mean ookinete density relative to pH8** | **SEM** |
| --- | --- | --- | --- | --- | --- | --- |
| pH 7.3 | - | 11 | 0.01 | 0.01 | 0.02 | 0.01 |
| pH 8 | - | 11 | 1.00 | 0.00 | 1.00 | 0.00 |
| KA | 0.000001 | 10 | 0.10 | 0.03 | 0.07 | 0.03 |
| KA | 0.00001 | 11 | 0.06 | 0.03 | 0.04 | 0.02 |
| KA | 0.0001 | 11 | 0.21 | 0.08 | 0.05 | 0.02 |
| KA | 0.001 | 11 | 0.28 | 0.11 | 0.21 | 0.06 |
| KA | 0.01 | 11 | 0.33 | 0.17 | 0.10 | 0.05 |
| KA | 0.1 | 6 | 0.00 | 0.00 | 0.00 | 0.00 |
| Tryp | 0.000001 | 11 | 0.14 | 0.10 | 0.04 | 0.02 |
| Tryp | 0.00001 | 11 | 0.08 | 0.05 | 0.03 | 0.01 |
| Tryp | 0.0001 | 11 | 0.11 | 0.07 | 0.04 | 0.02 |
| Tryp | 0.001 | 11 | 0.03 | 0.01 | 0.03 | 0.02 |
| Tryp | 0.01 | 11 | 0.04 | 0.02 | 0.03 | 0.01 |
| Tryp | 0.1 | 6 | 0.00 | 0.00 | 0.00 | 0.00 |
| XA | 0.000001 | 11 | 0.20 | 0.10 | 0.08 | 0.04 |
| XA | 0.00001 | 11 | 1.70 | 1.16 | 0.53 | 0.14 |
| XA | 0.0001 | 11 | 1.17 | 0.65 | 0.63 | 0.14 |
| XA | 0.001 | 11 | 1.18 | 0.53 | 0.78 | 0.12 |
| XA | 0.01 | 11 | 0.01 | 0.00 | 0.00 | 0.00 |
| XA | 0.1 | 6 | 0.00 | 0.00 | 0.00 | 0.00 |

**Table 2. *P. yoelii*** raw data. These data were log2 +0.001 transformed prior to analysis, as displayed in figure 3a, and log2 transformed for figure 3b.

| **Sub-**  **species** | **Treatment** | **n** | **Mean exflagellation density relative to pH8** | **SEM** | **Mean ookinete density (ookinetes per ml blood)** | **SEM** |
| --- | --- | --- | --- | --- | --- | --- |
| Pyn | pH 7.3 | 3 | 0.00 | 0.00 | 0 | - |
| Pyn | pH 8 | 3 | 1.00 | 0.00 | 1.75x10^7^ | 1.22x10^7^ |
| Pyn | KA | 3 | 0.12 | 0.02 | 0 | - |
| Pyn | Tryp | 3 | 0.05 | 0.02 | 0 | - |
| Pyn | XA | 3 | 0.62 | 0.31 | 2.61x10^6^ | 2.44x10^6^ |
| Pys | pH 7.3 | 3 | 0.01 | 0.01 | 0 | - |
| Pys | pH8 | 3 | 1.00 | 0.00 | 0 | - |
| Pys | KA | 3 | 0.10 | 0.07 | 0 | - |
| Pys | Tryp | 3 | 0.02 | 0.02 | 0 | - |
| Pys | XA | 3 | 0.23 | 0.10 | 5.67x10^4^ | 5.67x10^4^ |
| Pyy | pH 7.3 | 4 | 0.00 | 0.00 | 0 | - |
| Pyy | pH 8 | 5 | 1.00 | 0.00 | 8.50x10^5^ | 7.32x10^5^ |
| Pyy | KA | 5 | 0.00 | 0.00 | 0 | - |
| Pyy | Tryp | 5 | 0.01 | 0.01 | 0 | - |
| Pyy | XA | 5 | 0.16 | 0.14 | 2.72x10^5^ | 1.98x10^5^ |
|  |  |  |  |  |  |  |

**Table 3. Microparticle density experiment** raw data. These data were log10 transformed as displayed in the main text Figure 4.

| **Strain** | **Media replenished?** | **Microparticle density (%)** | **n** | **Mean ookinete density per ml blood** | **SEM** |
| --- | --- | --- | --- | --- | --- |
| Pb ANKA | Y | 0 | 3 | 7.29x10^7^ | 2.20x10^7^ |
| Pb ANKA | Y | 2 | 3 | 6.33x10^7^ | 2.34x10^7^ |
| Pb ANKA | Y | 35 | 3 | 1.29x10^7^ | 3.79x10^6^ |
| Pb ANKA | Y | 60 | 4 | 6.05x10^6^ | 5.94x10^6^ |
| Pb ANKA | N | 0 | 6 | 8.80x10^6^ | 4.13x10^6^ |
| Pb ANKA | N | 2 | 6 | 8.17x10^6^ | 3.33x10^6^ |
| Pb ANKA | N | 35 | 6 | 3.93x10^6^ | 1.94x10^6^ |
| Pb ANKA | N | 60 | 6 | 1.30x10^6^ | 6.65x10^5^ |
| Pb 820 | Y | 0 | 3 | 7.57x10^7^ | 1.14x10^7^ |
| Pb 820 | Y | 2 | 4 | 4.76x10^7^ | 1.30x10^7^ |
| Pb 820 | Y | 35 | 5 | 2.43x10^7^ | 1.01x10^7^ |
| Pb 820 | Y | 60 | 5 | 3.91x10^6^ | 9.77x10^5^ |
| Pb 820 | N | 0 | 3 | 1.60x10^7^ | 6.23x10^6^ |
| Pb 820 | N | 2 | 3 | 1.05x10^7^ | 6.51x10^6^ |
| Pb 820 | N | 35 | 3 | 1.84x10^7^ | 2.54x10^6^ |
| Pb 820 | N | 60 | 4 | 1.84x10^6^ | 8.97x10^5^ |

**Table 4. Microgamete attraction experiment**, raw data.

|  | | **Mean proportion of male gametes at the interface** | | | |
| --- | --- | --- | --- | --- | --- |
|  | **n** | **START** | ***SEM*** | **END** | ***SEM*** |
| **RBCs** | 10 | 0.494 | 0.019 | 0.494 | 0.019 |
| **Asexual infected cells** | 13 | 0.494 | 0.021 | 0.477 | 0.012 |
| **Lysed asexual material** | 12 | 0.464 | 0.013 | 0.459 | 0.027 |
| **Female gametocytes** | 9 | 0.499 | 0.013 | 0.546 | 0.025 |
| **Lysed female material** | 10 | 0.511 | 0.017 | 0.516 | 0.024 |
